# Clinician's Update on the Benign, Premalignant, and Malignant Skin Tumours of the Vulva: The Dermatologist's View

**DOI:** 10.1155/2017/2414569

**Published:** 2017-07-25

**Authors:** Freja Lærke Sand, Simon Francis Thomsen

**Affiliations:** ^1^Department of Dermatology, Bispebjerg Hospital, Copenhagen, Denmark; ^2^Department of Biomedical Sciences, University of Copenhagen, Copenhagen, Denmark

## Abstract

Correct and rapid diagnosis of skin tumours often requires biopsy and histopathological examination to differentiate benign lesions such as seborrhoeic keratoses or melanocytic naevi from premalignant and malignant lesions such as malignant melanoma. Particularly, to the untrained eye, any benign skin tumour—pigmented or nonpigmented—is easily mistaken for a malignant lesion. Qualified clinical evaluation is paramount in order to reduce the frequency of unwarranted skin biopsies. Herein, the most common benign, premalignant, and malignant vulvar skin tumours are reviewed.

## 1. Introduction

A variety of vulvar tumours are seen in daily clinical practice and the vast majority are benign. However, correct and rapid diagnosis often requires biopsy and histopathological examination in order to differentiate benign lesions such as seborrhoeic keratoses or melanocytic naevi from premalignant and malignant lesions such as malignant melanoma. Herein, we review the most common benign, premalignant, and malignant vulvar skin tumours.

## 2. Benign Tumours

### 2.1. Vulvar Squamous Papillomatosis

**Figure 1:**
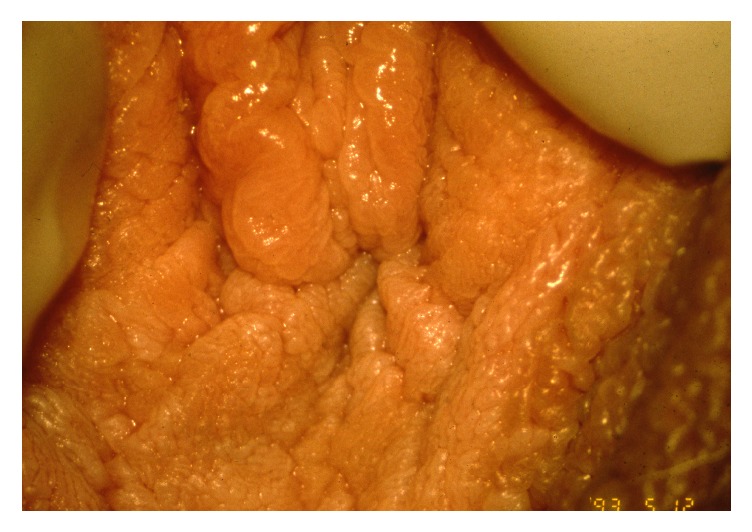
Squamous papillomatosis.

Multiple squamous papillae of the vulva are a benign normal variant of the vestibule in young women often mistaken for disseminated genital warts. Wart remedies such as podophyllotoxin and imiquimod, cryotherapy, and laser surgery may have been applied without benefit. The condition is asymptomatic and previous reports of vulvar squamous papillomatosis as a cause of concomitant pruritus and vulvodynia/vestibulodynia have not been substantiated [1, 2]. The clinical presentation is multiple prominent teardrop and rod-shaped papillae in the inner aspects of the labia (Figure 1). Histopathology shows prominent fibrovascular cores covered by mature squamous epithelium. Active treatment is not indicated.

### 2.2. Seborrhoeic Keratosis

**Figure 2:**
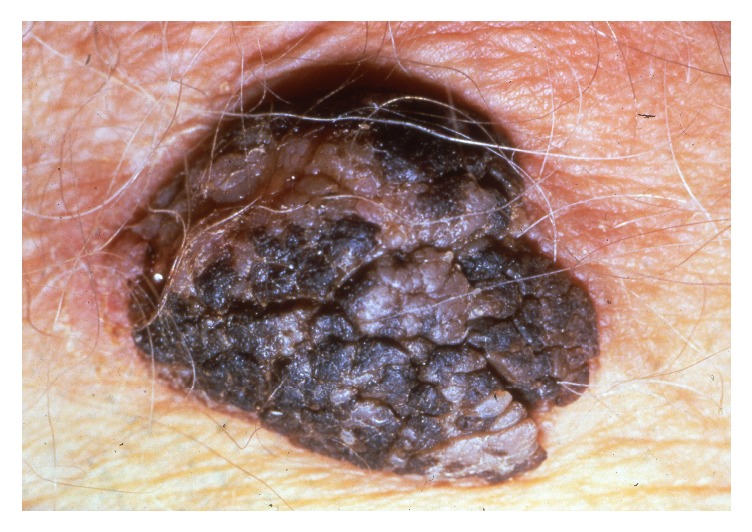
Seborrhoeic keratosis.

A seborrhoeic keratosis is a common benign keratinocyte neoplasm that in elderly women may be located in the vulvar area. The tumour is brown-black (but may be skin coloured in its early developmental stages), sharply delineated, smooth or verrucous, oval or round with a greasy texture, located on the keratinised skin in the vulvar area (Figure 2). Intensely pigmented lesions may mimic and be mistaken for a malignant melanoma [3]. Histopathology is characterised by a papillomatous acanthotic epithelium consisting of basal cells with small regular nuclei. There is hyperkeratosis with formation of pseudohorn cysts and melanin pigmentation may be prominent. Dermoscopy may be used in trained hands to differentiate highly pigmented seborrhoeic keratoses from malignant melanoma [3]. Therapy is curettage or cryosurgery with liquid nitrogen.

### 2.3. Epidermoid Cyst

**Figure 3:**
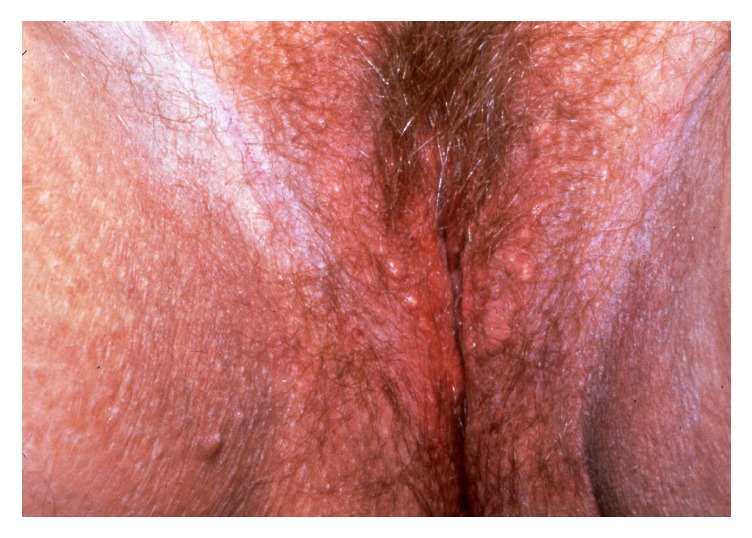
Epidermoid cysts.

Epidermoid cysts (atheromas) are 5–10 mm, smooth, dome-shaped, yellow-white lesions located on labia majora and around the clitoris in middle-aged and elderly women (Figure 3). Ruptured lesions may drain a thick greasy material. Giant vulvar epidermoid cysts may be encountered including lesions that may result in pseudoclitomegaly [4, 5]. Epidermoid cysts are unilocular, lined with flattened squamous epithelium containing laminated keratin. Secondary infection may result in a painful inflammatory reaction with rupture of the cyst wall. Small lesions may be removed by electrocautery or laser surgery, but larger lesions should be excised in toto.

### 2.4. Sebaceous Cyst

**Figure 4:**
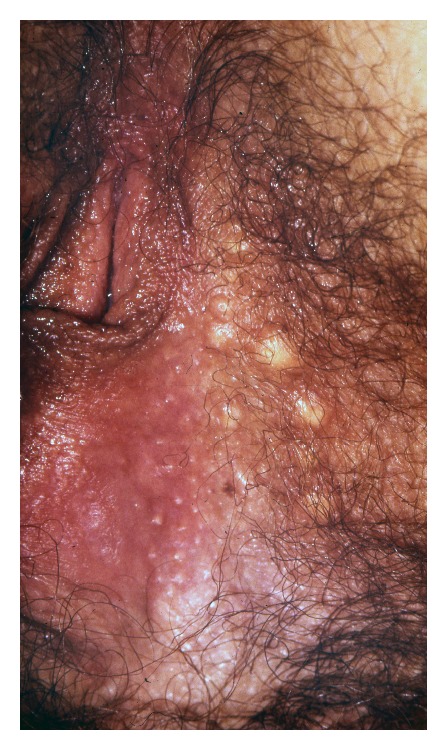
Sebaceous cysts.

Sebaceous cysts are caused by blocking of the duct of the multiple sebaceous glands of the hair-bearing surface of the vulva. Sebaceous retention cysts are usually asymptomatic small dome-shaped lesions with a translucent or yellowish colour that may contain a greasy yellow-white material (Figure 4) [6]. Occasionally, a lesion may present as a 2-3 cm large polypoid tumour of the vulva [7]. Women with steatocystoma multiplex, an autosomal inherited disease, have multiple sebaceous cysts in the axillae and femoral fold as well [8]. Histopathology is characterised by a thin-walled intraepidermal cyst lined with cells forming stratified nonkeratinised squamous epithelium. An inflammatory reaction may be seen if the cyst ruptures with the formation of a foreign body reaction (lipoid granuloma). Therapy is excision or in the case of inflammation intralesional triamcinolone may induce involution.

### 2.5. Bartholin Gland Cyst

Bartholin's glands located bilaterally at the posterior introitus drain through ducts that empty into the vestibule at the 4 and 8 o'clock positions and moisten the vulvar area. If the duct is obstructed, it may result in a unilateral painless small cystic swelling and palpable mass in the posterior commissure [9, 10]. Small cysts may be asymptomatic but larger cysts may cause discomfort when walking or sitting or during intercourse. The cyst is lined by columnar squamous or flattened epithelium and acini are present within the cyst wall. A small cyst may resolve spontaneously, whereas larger cysts may be treated with either catheterisation or marsupialisation. In recurrent cases, excision of Bartholin's gland is the best option.

### 2.6. Mucinous Cyst

Multiple mucinous glands of urogenital sinus origin are present during the development of the vulva. Small cysts may arise as a result of obstruction of the neck of these glands resulting in mucinous cysts located in the vulvar vestibule [11, 12]. A larger vulvar mucinous cyst is often a Bartholin's gland cyst when reviewed histopathologically after excision [13]. The cysts are lined with columnar epithelium. Therapy is usually not indicated as the cysts are asymptomatic.

### 2.7. Mesonephric Cyst

Mesonephric cysts, also termed Gardner's duct cysts, are located on the lateral aspects of the vulva and develop from mesonephric remnants [14]. The epithelial lining of the cyst is usually cuboidal. Therapy is rarely indicated, but larger cysts may be excised.

### 2.8. Milia

Milia are minute superficial epidermoid cysts lined with epidermis and filled with keratin that are thought to result from pilosebaceous or eccrine sweat duct plugging. Milia may be primary or secondary, the latter resulting from injury to the skin. Mila are 1-2 mm, white, domed-shaped papules usually located at the cheeks and eyelids of adults, but milia may also affect the vulvar area [15, 16]. Due to cosmetic reasons, lesions may be removed with electrodissection or expression of the keratin contents after incision.

### 2.9. Haemangioma

**Figure 5:**
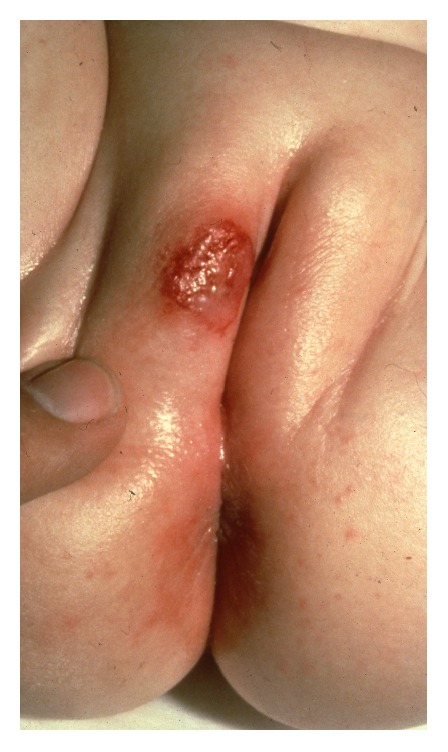
Infantile haemangioma.

Infantile capillary and cavernous haemangiomas are common lesions in newborns and may arise at any cutaneous site including the vulvar area. The lesion is a flat erythematous plaque that over months progresses to a nodular vascular tumour (Figure 5). Haemangiomas are rare in adult women, but large cavernous vulvar lesions have been described, including lesion causing clitomegaly [17, 18]. The diagnosis is clinical in infantile haemangiomas but biopsy and histopathology may be needed in adults with a vascular lesion to exclude endometriosis [19]. Infantile haemangiomas usually involute spontaneously over years, whereas this is not the case in adults with vulvar haemangiomas. Therapy in infantile haemangiomas is rarely indicated, but in children with giant lesions or lesions interfering with urination/defecation or in case of a painful ulcerating lesion, therapy with oral propranolol should be considered [20]. In adults with large haemangiomas, excision may be the treatment of choice. In rare instances, infantile haemangiomas may be associated with internal malformations.

### 2.10. Pyogenic Granuloma

A pyogenic granuloma is common benign vascular proliferation of the skin and mucous membranes that occasionally is located in the vulvar area [21, 22]. Minor trauma may cause excessive local production of angiogenic factors, which are suggested to be a pathogenic factor. Children and pregnant women are predisposed to developing pyogenic granulomas. A solitary sessile or pedunculated erythematous lesion that easily bleeds after minor trauma is the rule, but multiple exophytic vulvar lesions have been described [23]. Treatment is curettage in combination with electrocautery, CO_2_, or dye laser therapy.

### 2.11. Angiokeratoma

**Figure 6:**
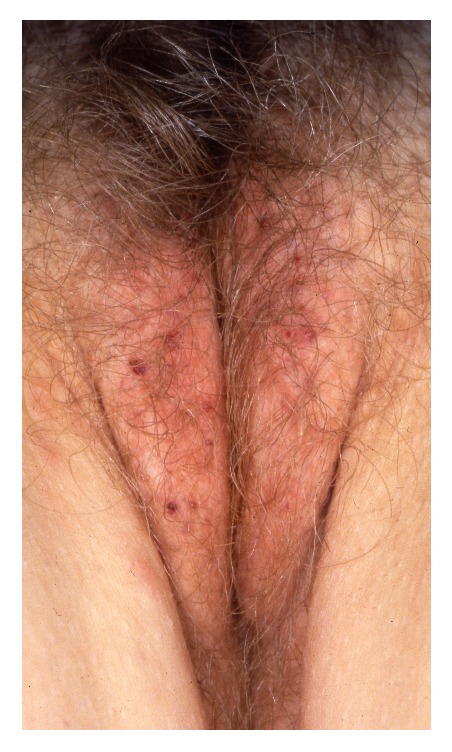
Angiokeratoma.

Angiokeratomas are vascular tumours consisting of numerous ectatic blood vessels in the superficial dermis. Angiokeratomas are seen in middle-aged and older women presenting with multiple asymptomatic erythematous or purple papules in the vulvar area (Figure 6) [24, 25]. Histopathology is characterised by ectatic vascular channels superficially in the papillary dermis surrounded by elongated dermal papillae. Angiokeratomas usually require no therapy, but treatment with electrocautery or pulsed dye laser may be effective [26].

### 2.12. Lymphangioma

**Figure 7:**
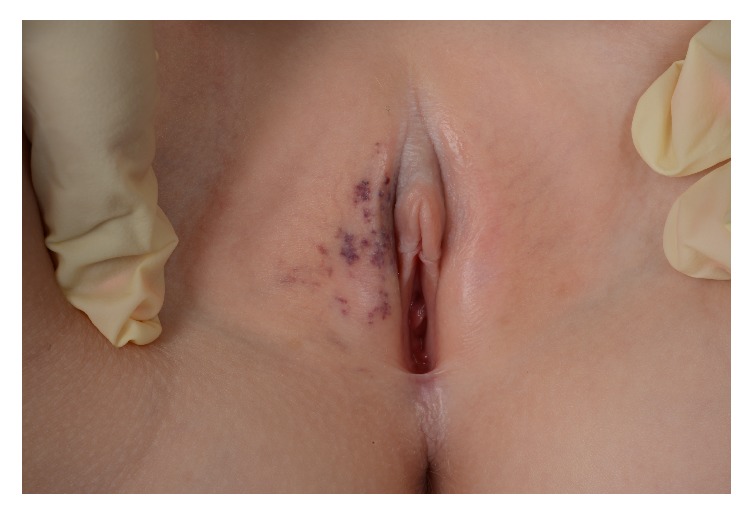
Lymphangioma.

Primary lymphangiomas or lymphangiomata circumscripta are rare, usually congenital lymphatic malformations diagnosed during infancy, whereas secondary or acquired lymphangiomas arise in adulthood as sequelae to surgery, radiotherapy, or other traumatising events. A vulvar lesion is characterised by frog egg-like confluent grouped thin-walled vesicles filled with serosanguineous fluid (Figure 7) [27]. Histopathologically, the lesions consist of dilated lymphatic channels with a flat endothelium. Therapy is difficult as recurrences are common irrespective of the treatment modality, which is why a conservative approach should be considered. Management options include surgical excision, laser, or sclerotherapy [27].

### 2.13. Syringoma

**Figure 8:**
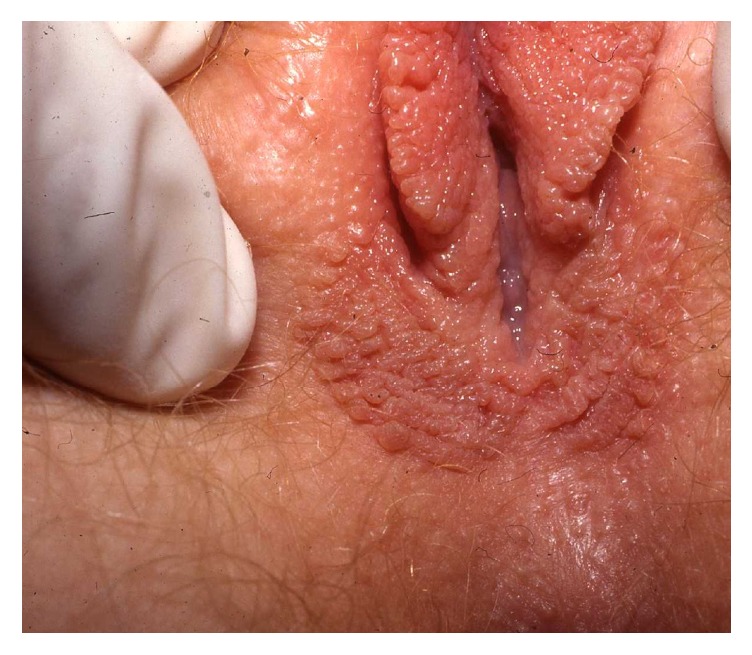
Syringoma.

A syringoma is a common tumour of eccrine sweat glands usually located on the lower eyelids, neck, chest, axillae, and genital area, including the vulva [28]. They emerge in early adult life with multiple small (1–3 mm), firm, skin-coloured or brownish papules bilaterally to the vulva (Figure 8). Vulvar lesions are usually asymptomatic but pruritus may be prominent in a subgroup of women. Pruritus may be aggravated by pregnancy [29]. Histopathology shows small strands of cells and ducts in a fibrous stroma within the dermis. The ducts are lined by two layers of cuboidal cells. Therapy should be restricted to women with intense pruritus and CO_2_ laser may be effective [30].

### 2.14. Schwannoma

Schwannomas or neuromas are benign encapsulated nerve sheath neoplasms that occasionally may be located in the vulvar area in adults [31]. The clinical presentation is a solitary nodular lesion. Histopathology shows neoplastic proliferation of Schwann cells. Surgical excision is the treatment of choice.

### 2.15. Leiomyoma

A leiomyoma is a rare benign neoplasm in adults derived from labial muscle presenting as a solitary flesh-coloured or reddish-brown, occasionally painful, nodule. The lesions range in size from a few mm to 1 cm [32]. Histopathology shows abundant smooth muscle fibres forming a solid tumour with intervening collagen. Malignant transformation to leiomyosarcoma is rare [32]. Therapy is excision.

### 2.16. Lipoma

Lipomas are common soft tissue tumours that can be located anywhere on the body, including the vulvar area [33]. Vulvar lipomas may also be seen in children [34]. The clinical presentation is a soft, painless, slowly growing vulvar nodule. Histopathology is characterised by a circumscribed tumour composed of mature adipocytes. Therapy is excision.

### 2.17. Hidradenoma Papilliferum

Hidradenoma papilliferum is an uncommon benign cutaneous adnexal tumour arising from apocrine sweat glands with predilection to the vulvar area in middle-aged women [35, 36]. It usually presents as a firm, well-circumscribed, skin-coloured, or erythematous cystic nodule measuring 1-2 cm in size, occasionally larger [36]. Histopathology reveals a tumour with tubular and papillary proliferation and cystic dilated spaces lined with columnar cells in the surface layer and cuboidal epithelium in the basal layer and usually with an outer layer of myoepithelial cells. Malignant transformation has been described in chronic cases [36]. Therapy is excision.

### 2.18. Endometriosis

The vulva is an uncommon site for endometriosis; however, the diagnosis should be considered in women with a persistent tender erythematous-bluish nodule usually located at the labia majora [37, 38]. It is associated with dyspareunia and increase of the lesion during menstruation. Histopathology shows endometrial stroma and glands with variable amounts of fibrosis and haemosiderosis. Therapy is excision.

### 2.19. Melanosis

**Figure 9:**
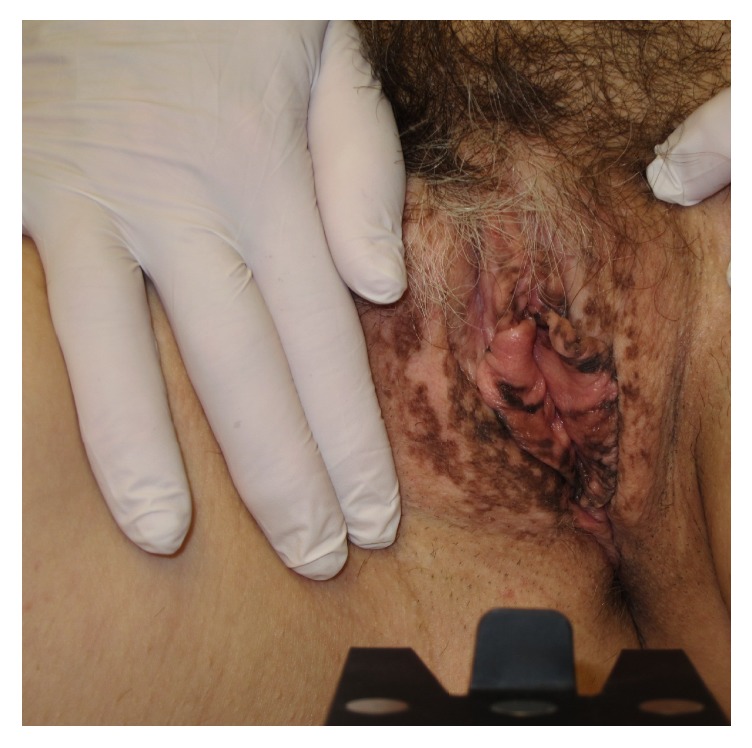
Melanosis.

Vulvar melanosis or vulvar lentiginosis, also referred to as lentigo simplex, is the most common pigmented lesion in adult women, representing approximately 70% of all pigmented vulvar lesions [39, 40]. The aetiology is unknown. Vulvar melanosis presents as single or multiple small (1–5 mm) dark-brown to black macules or patches on the mucosal surfaces (Figure 9). Histopathology shows increased melanin and a normal or slightly increased number of melanocytes in the basal layer of the epidermis and increased pigmentation within macrophages (melanophages) in the papillary dermis [41]. Vulvar melanosis follows a benign course and the risk of malignant melanoma is not increased. Regular follow-up may be considered in selected patients with disseminated lesions or in women with vulva melanosis in advanced age [42]. Therapy is not indicated.

### 2.20. Melanocytic Naevi

**Figure 10:**
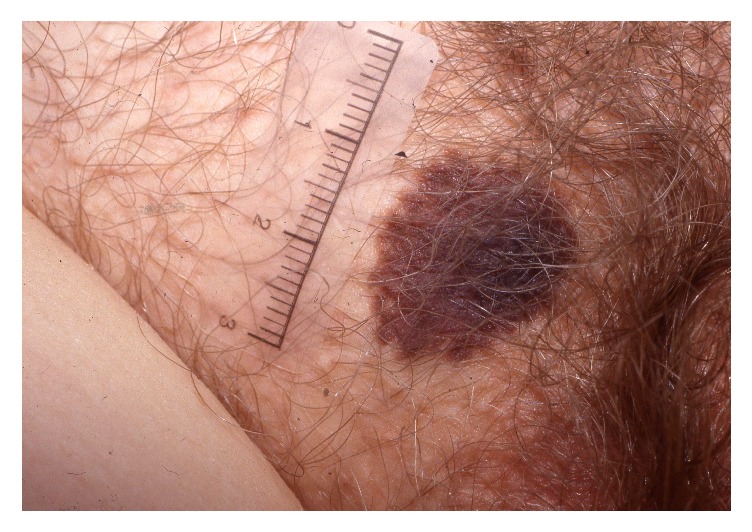
Melanocytic nevus.

Vulvar melanocytic naevi are seen in approximately 2% of adult premenopausal women and account for 23% of all pigmented lesions in this area [39, 40]. A similar number of children have vulvar melanocytic naevi [43]. A subgroup of younger women have “atypical melanocytic naevi of the genital type” (AMNGT) representing 5% of vulvar naevi [44]. These patients may have a personal or family history of dysplastic naevi or malignant melanoma [45]. Most vulvar naevi are pink to dark black-brown macules or flat-topped papules with well-demarcated borders, with uniform pigmentation, and with a diameter of less than 10 mm located on the labia majora, labia minora, and the clitoral hood (Figure 10). Blue macular naevi are occasionally seen. AMNGT may have darker pigmentation, irregular borders, and larger size. In trained hands, dermoscopy may assist in the diagnosis of vulvar naevi and AMNGT presenting with a predominant globular or homogeneous pigmentation pattern [46]. Histopathology shows groups of benign naevi cells in the basal epidermis, dermis, or both. Vulvar naevi have very low malignant potential as most (98%) of the malignant vulvar melanomas occur de novo [47]. Similarly, AMNGT have been found to possess low malignant potential [45, 48]. Therapy with excision is only indicated if malignancy is suspected.

## 3. Premalignant Tumours

### 3.1. Vulvar Intraepithelial Neoplasia

**Figure 11:**
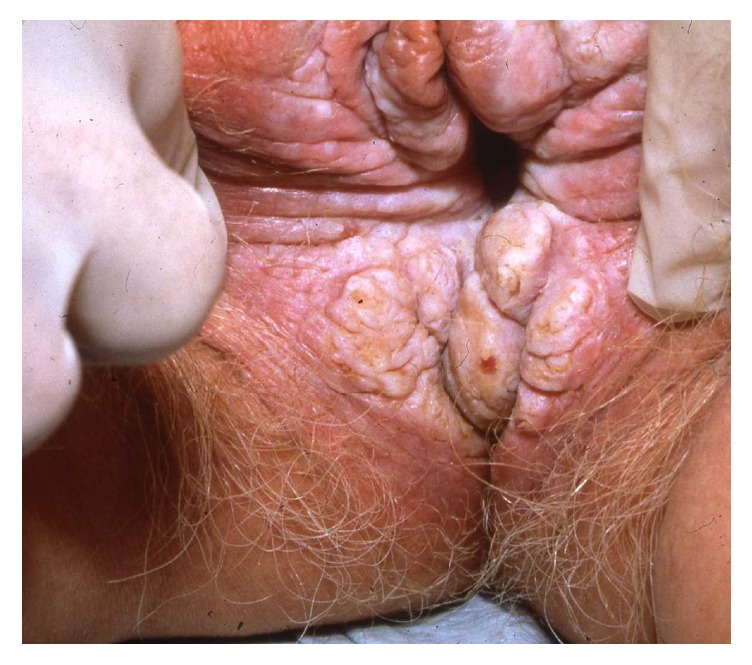
VIN in a woman with lichen sclerosus.

**Figure 12:**
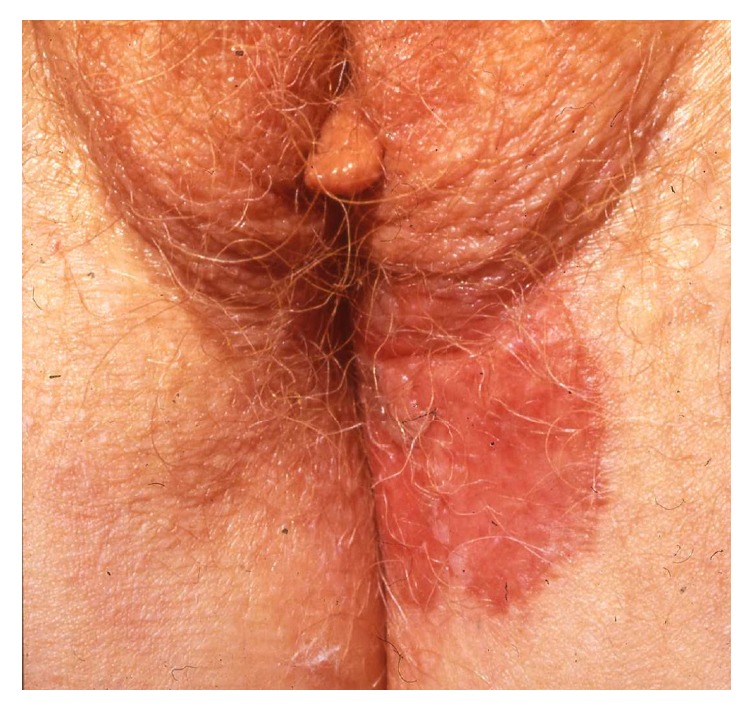
VIN with an erythematous plaque.

**Figure 13:**
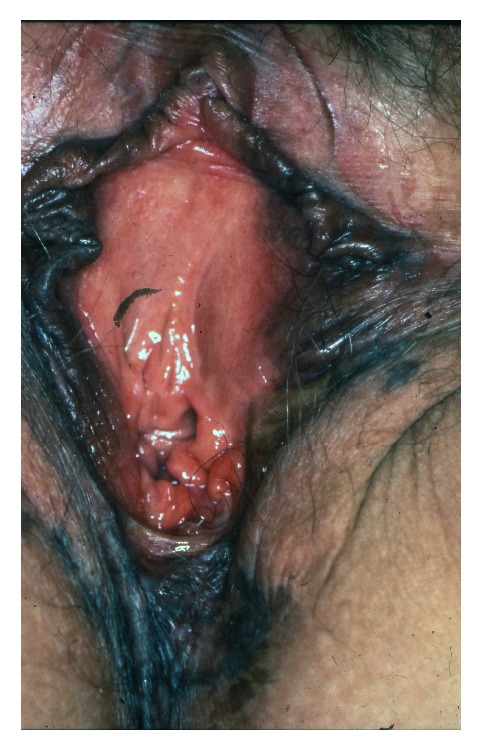
Pigmented VIN.

Vulvar intraepithelial neoplasia (VIN) is a high-grade intraepithelial precursor of invasive squamous cell carcinoma. Two different types of VIN have been defined: the common human papilloma virus (HPV) related type and the differentiated non-HPV-related type, the latter being associated with vulvar dermatoses, especially lichen sclerosus [49]. HPV-related VIN is the predominant clinical lesion (95%) with the highest frequency among younger women aged 20–35 years whereas the differentiated type of VIN (2–5%) most commonly occurs in elderly women [50]. The main HPV types involved in the pathogenesis of VIN are HPV 16, HPV 18, and HPV 33 [51]. In approximately 50% of women, VIN is asymptomatic. When symptomatic, the main complaints are pruritus, pain, and dyspareunia. The clinical findings are variable including unique or multiple flat, raised or eroded, white, erythematous or pigmented papules or plaques (Figures 11, 12, and 13). The differentiated type of VIN is usually unifocal, whereas the HPV type of VIN has a higher tendency to be multifocal and disseminated in the vulvar area.

The diagnosis should be confirmed by histopathology showing moderate to severe intraepithelial dysplasia; in the differentiated type of VIN, there are concomitant signs of lichen sclerosus or lichen simplex. Therapies of HPV-related VIN include surgical excision, CO_2_-laser ablation, photodynamic therapy, or topical treatment with imiquimod, 5-fluorouracil, or cidofovir [50, 52–54]. Surgical excision should be the first choice in women with the differentiated type of VIN due to the increased risk of malignant transformation. Irrespective of the treatment, the risk of recurrence is significant and long-term follow-up is mandatory. Similarly, women with vulvar VIN should be examined for concomitant cervical, vaginal, and intra-anal dysplasia often found in women with HPV-related VIN. The risk of malignant transformation of HPV-related VIN is lower (2.7% in younger women and 8.5% in elderly women) than for the differentiated type of VIN (33%) [55]. In general, immunosuppressed women have a higher risk of malignant transformation of VIN to invasive squamous cell carcinoma. On the other hand, spontaneous regression of VIN does occur in immune competent women.

HPV-related VIN due to HPV 16 and HPV 18 may be prevented by vaccination with the 4- or 2-valent vaccines [56]. A newly introduced 9-valent vaccine may also protect from VIN caused not only by HPV 16 and HPV 18, but also by HPV 31, HPV 33, HPV 45, HPV 52, and HPV 58 [57]. In addition, regular clinical examination of women with lichen sclerosus is recommended in order to recognise possible VIN in this high-risk group [58].

## 4. Malignant Tumours

### 4.1. Squamous Cell Carcinoma

**Figure 14:**
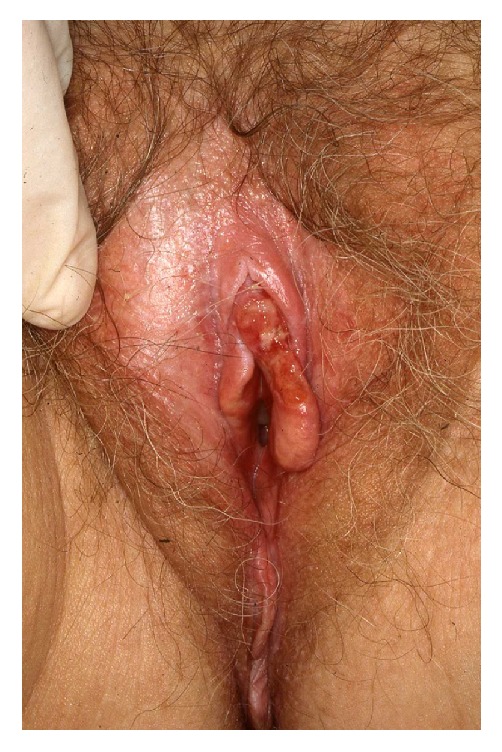
Squamous cell carcinoma.

Approximately 95% of malignant tumours of the vulva are squamous cell carcinoma (SCC), however only representing 5–10% of all gynaecological cancers [52]. Chronic vulvar HPV infection with one or more of the oncogenic HPV types is involved in half of the cases of SCC, whereas lichen sclerosus and to a lesser degree other chronic dermatoses, such as lichen planus, are predisposing factors in the non-HPV-related cases. Smoking may be an additional factor in HPV associated vulvar SCC. In the subset of women with the verrucous variant of SCC, the nononcogenic HPV types 6 and 11 may be a significant aetiological factor [59]. The incidence of both types of SCC (HPV and non-HPV associated) increases with age with a mean age at presentation of around 70 years, but HPV associated SCC may be seen in younger women and non-HPV associated SCC occasionally in younger women who have had lichen sclerosus since early childhood [55, 60, 61]. Vulvar SCC may appear as either an ulcerated endophytic lesion or a nodular exophytic tumour, most often located on labia majora or minora (Figure 14). Initial symptoms of pruritus, pain, and dyspareunia may be misdiagnosed as eczema and fungal infection and many women have been prescribed various topical therapies delaying correct diagnosis. The majority of vulvar SCC is solitary and only 10% is multifocal. At the time of diagnosis, approximately 20–30% has spread to regional lymph nodes [62]. The diagnosis is confirmed by histopathology showing irregular strands and cords of atypical squamous cells within the stroma. In the most common subtype, islands of keratinisation are present. Other histopathological subtypes include nonkeratinising SCC, basaloid carcinoma, warty carcinoma, verrucous carcinoma, SCC with tumour giant cells, and keratoacanthoma-like carcinoma.

Before therapy, the vulvar SCC should be staged according to the TNM system with extent of the carcinoma (T), whether the carcinoma has spread to lymph nodes (N) and whether it has spread to distant sites (M) [63]. In stage 1A with an invasion depth of less than 1 mm and size of less than 2 cm, the risk of nodal involvement is negligible. In stage 1B, stage II, and stage III, the risk of lymph node metastases increases to 10%, 25%, and 65%, respectively [63]. Early stage vulvar SCC should be excised with macroscopic tumour-free margins of 1 cm. Nodal staging is recommended in selected patients and if the tumour depth exceeds 1 mm with initial sentinel lymph node dissection and only radical inguinofemoral lymph node dissection if signs of metastases have been identified. MR- and PET-CT scans also add significant benefit in the staging of the tumour.

Local advanced disease with vaginal, urethral, or anal involvement can be treated with surgery combined with radiation therapy. The 5-year survival in women with radically excised local disease is 90%, but in case of metastases to regional lymph nodes survival decreases to 50% [62]. In nodal-negative patients with advanced local disease and recurrent disease, a 5-year survival rate of over 40% has been achieved [64].

### 4.2. Basal Cell Carcinoma

Basal cell carcinoma (BCC) is a common malignant tumour in elderly women usually located in UV exposed areas, but occasionally lesions occur in the vulvar area where it constitutes 2-3% of all vulvar cancers [65, 66]. HPV is not an aetiological factor in BCC [67]. The slowly growing tumour arises on labia majora with a nodular ulcerating lesion with rolled margins. The diagnosis is confirmed by histopathology usually showing irregular nests of basaloid cells with sparse cytoplasm and hyperchromatic nuclei within the dermis. Palisading of the most peripheral cells within the nest is common. However, the histopathological picture may deviate in the superficial and sclerosing types of BCC. Histopathologically, BCC should be differentiated from the HPV-related basaloid type of SCC. Therapy is excision with free margins. Recurrence is commonly seen if the margins initially were not sufficiently wide [65, 66]. Metastases do not occur.

### 4.3. Malignant Melanoma

Vulvar melanoma accounts for approximately 10% of all vulvar malignancies only exceeded by SCC [68]. An epidemiological study showed that 2.6% of all melanomas occurred in the vulvar region most commonly in older women [47]. Vulvar melanoma on the mucosal surface resembles the acral lentiginous type of melanoma rather than the cutaneous type of melanoma and ultraviolet radiation is not a significant factor in the pathogenesis. A vulvar melanoma is characterised by an asymmetrical black macule, papule, or nodule often with an irregular border and with a diameter larger than 7 mm located on the mucosal surface most frequently on the labia majora, labia minora, or the clitoral hood [47, 69]. Vulvar amelanotic melanoma is an erythematous lesion without the typical characteristics of melanoma which may imitate a pyogenic granuloma [70]. Dermoscopy may facilitate early identification of a melanoma suspect lesion with irregular dots and globules, multiple colours (black, blue, brown, pink, gray, and white), a blue-white veil, and atypical vessels [71]. The diagnosis is based on histopathology showing atypical melanocytes within the epidermis and dermis. The cells are arranged in confluent nests and sheets and contain varying amounts of melanin, and mitotic figures are usually abundant. The thickness of the lesion and the depth of invasion, ulceration, and lymph node involvement are important negative prognostic indicators. Therapy is wide excision or vulvectomy. In tumours thicker than 1 mm, sentinel node examination is recommended. Recently, immune therapy with monotherapy or combination therapy with one or more of the following drugs, ipilimumab, pembrolizumab, nivolumab, vemurafenib, dabrafenib, and trametinib, may be considered in women with disseminated melanoma. Due to late diagnosis, the prognosis of vulvar melanoma is poor with estimated 5-year survival ranging from 27 to 60% [72].

### 4.4. Vulvar Paget's Disease

**Figure 15:**
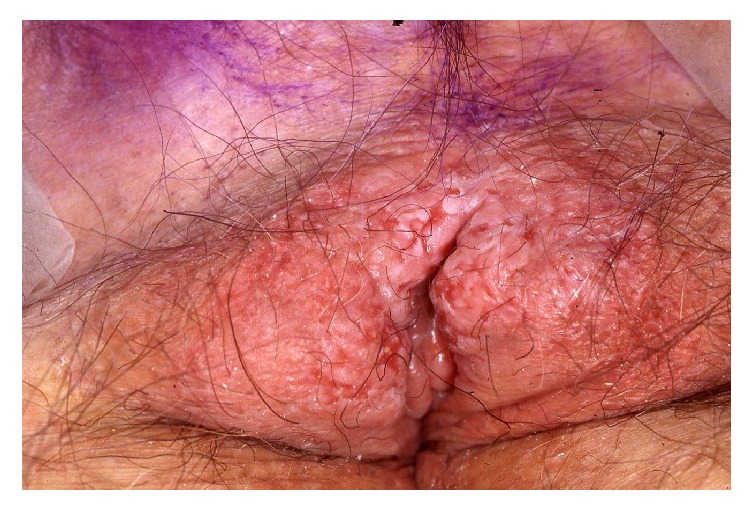
Paget's disease.

Although the vulva is the commonest site of extramammary Paget's disease, it only comprises 1-2% of vulvar malignancies [73]. Vulvar Paget's disease is a neoplastic condition representing an in situ intraepithelial adenocarcinoma derived from the intraepidermal component of apocrine sweat ducts in the majority of patients (75%) and in only 16% with concomitant dermal invasion [74]. In approximately 9%, vulvar Paget's disease is a manifestation of an underlying adenocarcinoma of dermal apocrine glands [74]. Finally, the lesion may also occasionally be due to epidermotropism from a more distant rectal, urogenital, or adenocarcinoma arising from elsewhere [75]. Vulvar Paget's disease affects elderly Caucasian women with a mean age of 65 years who over months develop a sharply demarcated pruritic or painful erythematous eczematoid plaque most often located at the labia majora and more rarely labia minora, introitus, and vagina (Figure 15). The size of the lesion varies from a few cm to an average of 6–12 cm [75]. Occasionally, the lesion may extend to the inguinal folds and the perianal region. A delay of the diagnosis is common and many have received corticosteroids and antifungals for months before a biopsy reveals vulvar Paget's disease. The diagnosis is based on histopathology demonstrating hyperplastic epidermis containing large cells with abundant pale-staining cytoplasm and large pleomorphic nuclei. The atypical cells contain mucin and express carcinoembryonic antigen. Surgical excision remains the mainstay of therapy despite the high risk of recurrence, probably because the visible borders do not correspond to the extent of histopathological involvement, which is why Mohs micrographic surgery is preferable [76]. In women with localised disease, a variety of other treatment modalities have been successful including topical imiquimod, 5-fluorouracil, laser surgery, and radio- and photodynamic therapy [75]. Combination of surgery with nonsurgical treatment may be used in selected cases [75]. Chemotherapy regimens have been used in women with metastatic Paget's disease [75].

### 4.5. Other Malignant Tumours

A variety of rare malignant tumours may arise in the vulvar area, many with a common clinical picture of a growing nodular lesion, which include sarcomas (epitheloid sarcoma, leiomyosarcoma, rhabdomyosarcoma, myxoid sarcoma, liposarcoma, dermatofibrosarcoma protuberans, and hemangiosarcoma) [77–81], adenocarcinomas (mucinous adenocarcinoma, eccrine hidradenocarcinoma) [82, 83], Merkel cell carcinoma [84], lymphomas, and metastases [85]. These vulvar malignant tumours may imitate benign lesions, but since many have metastatic potential rapid diagnosis is essential and biopsy and histopathologic examination of all vulvar lesions are of paramount importance. Therapy is primarily surgical with wide excision and groin lymphadenectomy in women with lymph node metastases after sentinel node technology [86].

## References

[1] Growdon W. A., Fu Y. S., Lebherz T. B., Rapkin A., Mason G. D., Parks G. (1985). Pruritic vulvar squamous papillomatosis: evidence for human papillomavirus etiology. *Obstetrics and Gynecology*.

[2] Manoharan V., Sommerville J. M. (1987). Benign squamous papillomatosis: case report. *BMJ Journals*.

[3] de Giorgi V., Massi D., Salvini C., Mannone F., Carli P. (2005). Pigmented seborrheic keratoses of the vulva clinically mimicking a malignant melanoma: a clinical, dermoscopic-pathologic case study. *Clinical and Experimental Dermatology*.

[4] Karaman E., Çim N., Akdemir Z., Elçi E., Akdeniz H. (2015). Giant vulvar epidermoid cyst in an adolescent girl. *Case Reports in Obstetrics and Gynecology*.

[5] Paulus Y. M., Wong A. E., Chen B., Jacobson M. T. (2010). Preputial epidermoid cyst: an atypical case of acquired pseudoclitoromegaly. *Journal of Lower Genital Tract Disease*.

[6] Sachdeva H. S., Narang R. (1968). Sebaceous cyst vulva. A case report.. *American Surgeon*.

[7] Ortiz-Rey J. A., Martín-Jiménez Á., Álvarez C., De La Fuente A. (2002). Sebaceous gland hyperplasia of the vulva. *Obstetrics & Gynecology*.

[8] Rongioletti F., Cattarini G., Romanelli P. (2002). Late onset vulvar steatocystoma multiplex. *Clinical and Experimental Dermatology*.

[9] Word B. (1968). Office treatment of cyst and abscess of bartholin's gland duct. *Southern Medical Journal*.

[10] Omole F., Simmons B. J., Hacker Y. (2003). Management of Bartholin's duct cyst and gland abscess. *American Family Physician*.

[11] Friedrich Jr. E. G., Wilkinson E. J. (1973). Mucous cysts of the vulvar vestibule. *Obstet Gynecol*.

[12] Robboy S. J., Ross J. S., Prat J., Keh P. C., Welch W. R. (1978). Urogenital sinus origin of mucinous and ciliated cysts of the vulva. *Obstetrics and Gynecology*.

[13] Rouzier R., Azarian M., Plantier F., Constancis E., Haddad B., Paniel B.-J. (2005). Unusual presentation of Bartholin's gland duct cysts: anterior expansions. *BJOG: An International Journal of Obstetrics and Gynaecology*.

[14] Janovski N. A., Weir J. H. (1962). Comparative histologic and histochemical studies of mesonephric derivatives and tumors. *Obstetrics and Gynecology*.

[15] Kanekura T., Kanda A., Higo A., Kanzaki T. (1996). Multiple milia localized to the vulva. *Journal of Dermatology*.

[16] Adotama P., Susa J., Glass D. A. (2014). Primary milia localized to the vulva. *Dermatology Online Journal*.

[17] Cebesoy F. B., Kutlar I., Aydin A. (2008). A rare mass formation of the vulva: giant cavernous hemangioma. *Journal of Lower Genital Tract Disease*.

[18] Bruni V., Pontello V., Dei M., Alessandrini M., Li Marzi V., Nicita G. (2009). Hemangioma of the clitoris presenting as clitoromegaly: a case report. *Journal of Pediatric and Adolescent Gynecology*.

[19] Papalas J. A., Sangueza O. P., Puri P. K., Robboy S. J., Selim M. A. (2013). Vulvar vascular tumors: a clinicopathologic study of 85 patients. *American Journal of Dermatopathology*.

[20] Léauté-Labréze C., Hoeger P., Mazereeuw-Hautier J., etal. (2015). A randomized controlled trial of oral propranolol in infantile hemangioma. *The New England Journal of Medicine*.

[21] Gupta S., Radotra B. D., Kumar B. (2000). Multiple, genital lobular capillary haemangioma (pyogenic granuloma) in a young woman: a diagnostic puzzle. *Sexually Transmitted Infections*.

[22] Patrizi A., Neri I., Guareschi E., Cocchi G. (2004). Multiple pyogenic granuloma involving female genitalia: a rare entity?. *Pediatric Dermatology*.

[23] Arikan D. C., Kiran G., Sayar H., Kostu B., Coskun A., Kiran H. (2011). Vulvar pyogenic granuloma in a postmenopausal woman: case report and review of the literature. *Case Reports in Medicine*.

[24] Blair C. (1970). Angiokeratoma of the vulva. *British Journal of Dermatology*.

[25] Terzakis E., Androutsopoulos G., Zygouris D., Grigoriadis C., Derdelis G., Arnogiannaki N. (2011). Angiokeratoma of the vulva. *European Journal of Gynaecological Oncology*.

[26] Oni G., Mahaffey P. (2010). Treatment of angiokeratoma of the vulva with pulsed dye laser therapy. *Journal of Cosmetic and Laser Therapy*.

[27] Vlastos A.-T., Malpica A., Follen M. (2003). Lymphangioma circumscriptum of the vulva: a review of the literature. *Obstetrics and Gynecology*.

[28] Carneiro S. J. C., Gardner H. L., Knox J. M. (1971). Syringoma of the Vulva. *Archives of Dermatology*.

[29] Bal N., Aslan E., Kayaselçuk F., Tarim E., Tuncer I. (2003). Vulvar syringoma aggravated by pregnancy. *Pathology & Oncology Research*.

[30] Huang Y.-H., Chuang Y.-H., Kuo T.-T., Yang L.-C., Hong H.-S. (2003). Vulvar syringoma: a clinicopathologic and immunohistologic study of 18 patients and results of treatment. *Journal of the American Academy of Dermatology*.

[31] Park S. T., Kim H. M., Shin M. K., Kim J. W. (2015). An unusual case of vulvar schwannoma. *World Journal of Surgical Oncology*.

[32] Nielsen G. P., Rosenberg A. E., Koerner F. C., Young R. H., Scully R. E. (1996). Smooth-muscle tumors of the vulva: a clinicopathological study of 25 cases and review of the literature. *The American Journal of Surgical Pathology*.

[33] Odoi A. T., Owusu-Bempah A., Dassah E. T., Darkey D. E., Quayson S. E. (2011). Vulvar lipoma: is it so rare?. *Ghana medical journal*.

[34] Oh J.-T., Choi S. H., Ahn S. G., Kim M. J., Yang W. I., Han S. J. (2009). Vulvar lipomas in children: an analysis of 7 cases. *Journal of Pediatric Surgery*.

[35] Kaufmann T., Pawl N. O., Soifer I., Greston W. M., Kleiner G. J. (1987). Cystic papillary hidradenoma of the vulva: case report and review of the literature. *Gynecologic Oncology*.

[36] Duhan N., Kalra R., Singh S., Rajotia N. (2011). Hidradenoma papilliferum of the vulva: case report and review of literature. *Archives of Gynecology and Obstetrics*.

[37] Brug P., Gueye N.-A., Bachmann G. (2012). Vulvar endometriosis presenting with dyspareunia: a case report. *Journal of Reproductive Medicine*.

[38] Abdullgaffar B., Keloth T. R., Raman L. G. (2014). Unusual benign polypoid and papular neoplasms and tumor-like lesions of the vulva. *Annals of Diagnostic Pathology*.

[39] Rock B., Hood A. F., Rock J. A. (1990). Prospective study of vulvar nevi. *Journal of the American Academy of Dermatology*.

[40] Rock B. (1992). Pigmented lesions of the vulva. *Dermatologic Clinics*.

[41] Lenane P., Keane C. O., Connell B. O., Loughlin S. O., Powell F. C. (2000). Genital melanotic macules: clinical, histologic, immunohistochemical, and ultrastructural features. *Journal of the American Academy of Dermatology*.

[42] Murzaku E. C., Penn L. A., Hale C. S., Pomeranz M. K., Polsky D. (2014). Vulvar nevi, melanosis, and melanoma: an epidemiologic, clinical, and histopathologic review. *Journal of the American Academy of Dermatology*.

[43] Hunt R. D., Orlow S. J., Schaffer J. V. (2014). Genital melanocytic nevi in children: experience in a pediatric dermatology practice. *Journal of the American Academy of Dermatology*.

[44] Ribé A. (2008). Melanocytic lesions of the genital area with attention given to atypical genital nevi. *Journal of Cutaneous Pathology*.

[45] Gleason B. C., Hirsch M. S., Nucci M. R. (2008). A typical genital nevi: a clinicopathologic analysis of 56 cases. *American Journal of Surgical Pathology*.

[46] Ferrari A., Zalaudek I., Argenziano G. (2011). Dermoscopy of pigmented lesions of the vulva: a retrospective morphological study. *Dermatology*.

[47] Ragnarsson-Olding B. K., Kanter-Lewensohn L. R., Lagerlöf B., Nilsson B. R., Ringborg U. K. (1999). Malignant melanoma of the vulva in a nationwide, 25-year study of 219 Swedish females: clinical observations and histopathologic features. *Cancer*.

[48] Clark Jr. W. H., Hood A. F., Tucker M. A., Jampel R. M. (1998). A typical melanocytic nevi of the genital type with a discussion of reciprocal parenchymal-stromal interactions in the biology of neoplasia. *Human Pathology*.

[49] Preti M., Scurry J., Marchitelli C. E., Micheletti L. (2014). Vulvar intraepithelial neoplasia. *Best Practice and Research: Clinical Obstetrics and Gynaecology*.

[50] Madsen B. S., Jensen H. L., van den Brule A. J. C., Wohlfahrt J., Frisch M. (2008). Risk factors for invasive squamous cell carcinoma of the vulva and vagina-population-based case-control study in Denmark. *International Journal of Cancer*.

[51] de Sanjose S., Alemany L., Ordi J. (2013). Worldwide human papillomavirus genotype attribution in over 2000 cases of intraepithelial and invasive lesions of the vulva. *European Journal of Cancer*.

[52] Léonard B., Kridelka F., Delbecque K. (2014). A clinical and pathological overview of vulvar condyloma acuminatum, intraepithelial neoplasia, and squamous cell carcinoma. *BioMed Research International*.

[53] Pepas L., Kaushik S., Bryant A., Nordin A., Dickinson H. O. (2011). Medical interventions for high grade vulval intraepithelial neoplasia. *Cochrane database of systematic reviews (Online)*.

[54] Tristram A., Hurt C. N., Madden T. (2014). Activity, safety, and feasibility of cidofovir and imiquimod for treatment of vulval intraepithelial neoplasia (RT3VIN): a multicentre, open-label, randomised, phase 2 trial. *The Lancet Oncology*.

[55] van de Nieuwenhof H. P., Massuger L. F. A. G., van der Avoort I. A. M. (2009). Vulvar squamous cell carcinoma development after diagnosis of VIN increases with age. *European Journal of Cancer*.

[56] Munoz N., Kjaer S. K., Sigurdsson K., Iversen O. E., Hernandez-Avila M., Wheeler et al. C. M. (2010). Impact of human papillomavirus (HPV)-6/11/16/18 vaccine on all HPV-associated genital diseases in young women. *Journal of the National Cancer Institute*.

[57] Joura E. A., Giuliano A. R., Iversen O. E. (2015). Broad spectrum HPV vaccine study. a 9-valent HPV vaccine against infection and intraepithelial neoplasia in women. *The New England Journal of Medicine*.

[58] MacLean A. B., Jones R. W., Scurry J., Neill S. (2009). Vulvar cancer and the need for awareness of precursor lesions. *Journal of Lower Genital Tract Disease*.

[59] Kondi-Paphitis A., Deligeorgi-Politi H., Liapis A., Plemenou-Frangou M. (1998). Human papilloma virus in verrucus carcinoma of the vulva: an immunopathological study of three cases. *European Journal of Gynaecological Oncology*.

[60] Saraiya M., Watson M., Wu X. (2008). Incidence of in situ and invasive vulvar cancer in the US, 1998-2003. *Cancer*.

[61] Carlson J. A., Ambros R., Malfetano J. (1998). Vulvar lichen sclerosus and squamous cell carcinoma: a cohort, case control, and investigational study with historical perspective; implications for chronic inflammation and sclerosis in the development of neoplasia. *Human Pathology*.

[62] Green Jr. T. H. (1978). Carcinoma of the vulva. A reassessment. *Obstet Gynecol*.

[63] Pecorelli S. (2009). Revised FIGO staging for carcinoma of the vulva, cervix, and endometrium. *International Journal of Gynecology and Obstetrics*.

[64] Fleisch M. C., Pantke P., Beckmann M. W. (2007). Predictors for interdisciplinary salvage surgery for advanced or recurrent gynecologic cancers. *Journal of Surgical Oncology*.

[65] Copas P., Spann Jr. C. O., Majmudar B., Horowitz I. R. (1996). Basal cell carcinoma of the vulva. A report of four cases. *The Journal of Reproductive Medicine*.

[66] Palladino V. S., Duffy J. L., Bures G. J. (1969). Basal cell carcinoma of the vulva. *Cancer*.

[67] Elwood H., Kim J., Yemelyanova A., Ronnett B. M., Taube J. M. (2014). Basal cell carcinomas of the vulva: high-risk human papillomavirus DNA detection, p16 and BerEP4 expression. *American Journal of Surgical Pathology*.

[68] Moxley K. M., Fader A. N., Rose P. G. (2011). Malignant melanoma of the vulva: an extension of cutaneous melanoma?. *Gynecologic Oncology*.

[69] Edwards L. (2010). Pigmented vulvar lesions. *Dermatologic Therapy*.

[70] Filippetti R., Pitocco R. (2015). Amelanotic vulvar melanoma: a case report. *American Journal of Dermatopathology*.

[71] Blum A., Simionescu O., Argenziano G. (2011). Dermoscopy of pigmented lesions of the mucosa and the mucocutaneous junction: Results of a multicenter study by the International Dermoscopy Society (IDS). *Archives of Dermatology*.

[72] Ragnarsson-Olding B. K. (2004). Primary malignant melanoma of the vulva: an aggressive tumor for modeling the genesis of non-UV light-associated melanomas. *Acta Oncologica*.

[73] Lloyd J., Flanagan A. M. (2000). Mammary and extramammary Paget's disease. *Journal of Clinical Pathology*.

[74] Niikura H., Yoshida H., Ito K. (2006). Paget's disease of the vulva: Clinicopathologic study of type 1 cases treated at a single institution. *International Journal of Gynecological Cancer*.

[75] Delport E. S. (2013). Extramammary Paget's disease of the vulva: an annotated review of the current literature. *Australasian Journal of Dermatology*.

[76] Tebes S., Cardosi R., Hoffman M. (2002). Paget's disease of the vulva. *American Journal of Obstetrics and Gynecology*.

[77] Chokoeva A. A., Tchernev G., Cardoso J. C. (2015). Vulvar sarcomas: short guideline for histopathological recognition and clinical management. Part 1. *International Journal of Immunopathology and Pharmacology*.

[78] Chokoeva A. A., Tchernev G., Cardoso J. C. (2015). Vulvar sarcomas: short guideline for histopathological recognition and clinical management. Part 2. *International Journal of Immunopathology and Pharmacology*.

[79] González-Bugatto F., Añón-Requena M. J., López-Guerrero M. A., Báez-Perea J. M., Bartha J. L., Hervías-Vivancos B. (2009). Vulvar leiomyosarcoma in Bartholin's gland area: a case report and literature review. *Archives of Gynecology and Obstetrics*.

[80] Gilani S., Al-Khafaji B. (2014). Dermatofibrosarcoma protuberans of the vulva: a mesenchymal tumour with a broad differential diagnosis and review of literature. *Pathologica*.

[81] Altundag K., Dikbas O., Oyan B., Usubutun A., Turker A. (2004). Epithelioid sarcoma of vulva: a case report and review of the literature. *Medical Oncology*.

[82] Massad L. S., Bitterman P., Clarke-Pearson D. L. (1996). Metastatic clear cell eccrine hidradenocarcinoma of the vulva: survival after primary surgical resection. *Gynecologic Oncology*.

[83] Tang Q., Jiang X., Lin Z., Li H., Wang Y., Luo X. (2011). Primary adenoid cystic carcinoma of sweat glands in vulva: report of an unusual case and review of the literature. *Journal of Obstetrics and Gynaecology Research*.

[84] Jońska-Gmyrek J., Bobkiewicz P., Gmyrek L., Żółciak-Siwińska A., Lindner B., Staniaszek J. (2013). Merkel cell carcinoma of the vulva – case report and the literature review. *Ginekologia Polska*.

[85] Neto A. G., Deavers M. T., Silva E. G., Malpica A. (2003). Metastatic tumors of the vulva: a clinicopathologic study of 66 cases. *American Journal of Surgical Pathology*.

[86] Crosbie E. J., Slade R. J., Ahmed A. S. (2009). The management of vulval cancer. *Cancer Treatment Reviews*.

